# Early prediction of acute kidney injury after liver transplantation by scoring system and decision tree

**DOI:** 10.1080/0886022X.2021.1945462

**Published:** 2021-07-14

**Authors:** Wang Xin, Wang Yi, Hui Liu, Liu Haixia, Lin Dongdong, Yingmin Ma, Guangming Li

**Affiliations:** a Department of Intensive Medicine, Beijing Youan Hospital, Capital Medical University, Beijing, China; b Department of Pharmacy, Beijing Haidian Hospital, Beijing, China; c Department of General Surgery, Beijing Youan Hospital, Capital Medical University, Beijing, China; d Department of Respiratory and Infectious Diseases, Beijing Youan Hospital, Capital Medical University, Beijing, China

**Keywords:** AKI after LT, early detection, scoring system, decision tree

## Abstract

**Background and aims:**

Early detection of acute kidney injury (AKI) is crucial for the prognosis of patients after liver transplantation (LT). This passage aims to analyze the perioperative clinical markers of AKI after LT and establish predictive models based on clinical variables for early detection of AKI after LT.

**Methods:**

We prospectively collected 109 patients with LT, and compared the differences of perioperative clinical markers between the AKI group and non-AKI group. The scoring system and decision tree model were established through the risk factors. Another 163 patients who underwent LT in the same center from 2017 to 2018 were retrospectively collected to verify the models.

**Results:**

In multiple comparisons of risk factors of post-LT AKI, pre-operative factors were excluded automatically, intraoperative and post-operative factors including operating time, intraoperative hypotension time, post-operative infection, the peak of post-operative AST, and post-operative shock were the independent risk factors for post-LT AKI. The scoring system established with the risk factors has good predictive power (AUC = 0.755) in the validation cohort. The decision tree also shows that post-operative shock was the most important marker, followed by post-operative infection.

**Conclusion:**

Five intraoperative and post-operative factors are independently associated with post-LT AKI rather than pre-operative factors, which indicates that operation technique and post-operative management may more important for the prevention of post-LT AKI. The scoring system and decision tree model could complement each other, and provide quantitative and intuitive prediction tools for clinical practice of early detection of post-LT AKI.

## 1. Introduction

Acute kidney injury (AKI) is commonly seen in critically ill patients in ICU [1], it is also one of the most common complications after liver transplantation (LT), and its mechanism has not been fully elucidated. In recent years, with the advancement of surgical techniques, the short-term and long-term survival rates of patients after LT have increased significantly. However, the occurrence of AKI after LT (post-LT AKI) is still one of the important reasons for the poor prognosis with morbidity of over 50% [2,3], compared with non-AKI, post-operative AKI will prolong the hospitalization, and increase the rates of acute rejection and infectious complications [4–7]. Previous researches showed that reducing the use of nephrotoxic drugs during the perioperative period and early intervention, such as renal replacement therapy (RRT) when necessary in the post-operative period could downgrade AKI after LT and improve the prognosis of AKI patients [8,9]. Therefore, early prevention of some risk factors and early detection of AKI risk is critical for patients after LT.

The development of post-LT AKI proved to be multifactorial, including pre-operative, intraoperative, and post-operative factors [10]. In previous studies, prediction models about post-LT AKI usually include only the pre-operative and intraoperative factors, but with rarely post-operative factors [11]. However, our previous study found that some post-operative factors are important for the development of AKI, such as post-operative liver function status, post-operative infection, and the application of immunosuppressive agents with renal toxicity, etc. Therefore in this study, we analyzed the perioperative risk factors of AKI, and included pre-operative, intraoperative, and post-operative indicators at the same time, aiming at establishing the effective scoring system and decision tree model for post-LT AKI.

## 2. Materials and methods

### 2.1. Participants

This study continuously collected the clinical data of all patients who underwent LT in the general surgery department of Beijing Youan Hospital throughout 2019. After excluding five patients with a history of chronic kidney diseases (CKD), such as IgA nephropathy before LT, a total of 109 patients were enrolled in the study as a model group. We also retrospectively collected some of the data (based on the marker in multiple analysis) of all patients underwent LT in the same center from January 2017 to December 2018. After excluding nine patients with CKD, the remaining 163 cases were used as a validation group. In the total of 272 cases, there are 229 males and 43 females with an average age of 53 years, ranges from 26 to 74 years. The diagnosis and classification criteria for AKI are based on the Kidney Disease Improving Global Outcomes (KDIGO) 2012 guidelines. The study was approved by the Ethical Committee of Beijing Youan Hospital (protocol number [2019] 016). Informed consents were signed by recipients and their families in 2019 and were waived in retrospective data during 2017–2018. The clinical study registration number is CHiCTR1900024561 in http://www.chictr.org/cn/.

### 2.2. Diagnosis criteria

AKI was defined as a syndrome from those patients who developed mild-to-severe AKI within 1 month after LT, which was defined using the KDIGO 2012 guidelines [12]: (1) an increase in serum creatinine (Scr) by ≥26.5 μmol/L within 48 h; (2)an increase in Scr to ≥1.5 times baseline within the first 7 post-operative days; (3)the urine volume (UV) <0.5 mL/(kg·h) lasted more than 6 h after LT. Moreover, AKI was classified into three stages: stage 1, creatinine increase to ≥26.5 μmol/L or increase to 1.5–1.9-fold from baseline, or the UV <0.5 mL/(kg·h) lasted for 6–12 h; stage 2, increase to 2–2.9-fold or UV <0.5 mL/(kg·h) lasted >12 h; stage 3, increase >3-fold or increase in Scr to ≥354 μmol/L or initiation of CRRT. The baseline Scr was defined as the lowest creatinine within 1 month before transplantation. Patients who had a history of CKD before transplantation were excluded from the study.

### 2.3. Data Collection

This study collected the demographic data of the recipient cases. Based on the previous literature, the data related to perioperative variables known to be related to post-transplant renal dysfunction were collected from the institutional electronic medical record. Demographic data include gender, age, and BMI. Other pre-operative factors include primary liver disease and other primary diseases. Primary liver diseases include acute/subacute liver failure with absolute priority, decompensated liver cirrhosis (model for end stage liver disease (MELD) scores ≥15), and patients with hepatoma who meet the ‘Milan Criteria’ [13]. The perioperative clinical markers are divided into pre-operative, intraoperative, and post-operative factors. Among them, pre-operative markers include Child-Pugh classification, MELD scores [14], serum biochemical parameters, pre-operative infection, hepatorenal syndrome, etc.; Surgical and anesthesia-related markers include fatty liver of donor, surgical technique, operation time, anhepatic phase, cold ischemia time of donor's liver, intraoperative bleeding and blood transfusion volume, intraoperative hypotension status and duration (intraoperative hypotension standard: systolic blood pressure <90 mmHg or decreases >20% of the baseline), intraoperative fluid balance and intraoperative urine, etc.; post-transplant indicators include the lactic acid immediately after the operation, serum biochemical parameters within 7 days after surgery, early allograft dysfunction (EAD) [15], acute respiratory distress syndrome (ARDS), mechanical ventilation time, shock, infection and severe infections, etc. [16]. According to the consensus of experts on sepsis, infection was defined as SIRS with a presumed or confirmed infectious process, and severe infection was defined as infection with ≥1 sign of organ failure. Moreover, post-operative shock was defined as any cause in the patient whose systolic blood pressure (SBP) <90 mmHg, or SBP was lower than the baseline blood pressure (BP) by more than 40 mmHg with a history of hypertension, or the shock index (pulse rate/SBP) ≥1, when there was an incentive for shock (such as severe infection, post-operative bleeding, and severe underlying heart disease, etc.). Univariate and multiple analysis of the above indicators were conducted to find out the risk factors of AKI after LT and then used to establish predictive algorithm models.

### 2.4. Construction and model validation

Decision tree (also called classification and regression tree, CART) model: the factors associated with AKI after LT were analyzed with Chi-squared automatic interaction detector (CHAID) algorithm. Classification rules: the growing ‘branch’ split test level is at αmerge + αsplit = 0.05. The minimum sample size of the parent node is 30, and the child node is 10. Multiple logistic regression model: factors associated with AKI were analyzed with the stepwise backward screening method. Scoring system based on logistic regression (imitated the modeling method by Framingham Heart Study) [17]: the continuous variables were firstly stratified according to the clinical significance or usage habits. And for each factor, an appropriate group was chosen as the reference value, and assign 0 points. Combining the regression coefficients in logistic analysis, the distance between each stratification and the reference value was calculated, and each stratification was further assigned. And then, the range of scores was calculated. Finally, according to the equations of the logistic regression model, the risk value corresponding to each score was calculated for prediction. The calculation formula is:

$$\hat{p}=\frac{1}{1+\;\text{exp}\;\left(-\sum_{i=0}^{p}\beta_{i}X_{i}\right)}$$

**Figure 1.**
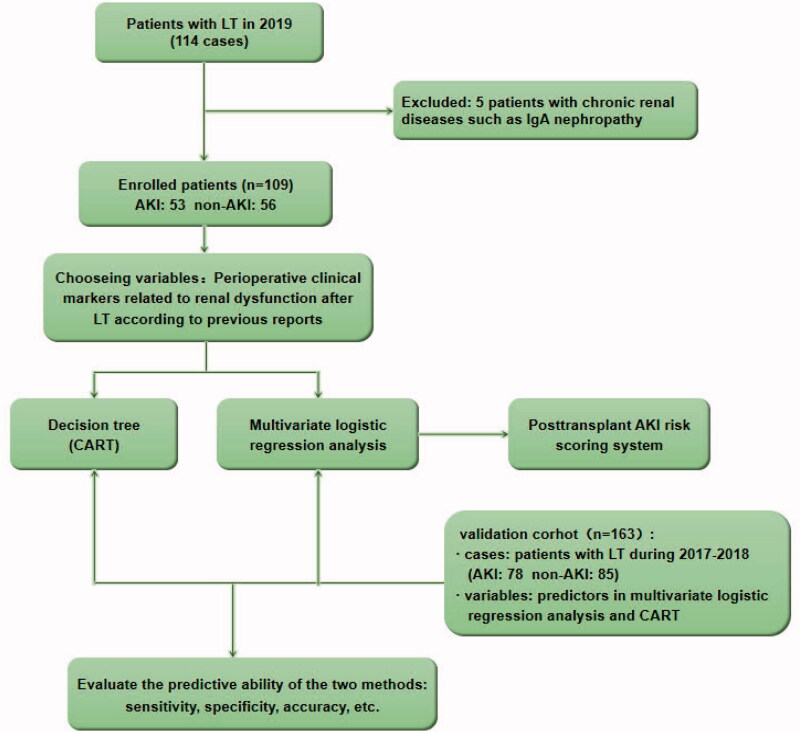
Flowchart of the inclusion and exclusion criteria of the study population. LT: liver transplant; AKI: acute kidney injury.

During model validation, ROC curves were used to evaluate the sensitivity, specificity, and accuracy of the CART model and the logistics regression model in the validation group. Figure 1 shows the exclusion criteria and the research design.

### 2.5. Statistical analysis

Normally distributed enumeration variables were described as mean ± standard deviation (*SD*), and *t*-test was used for comparisons. Non-normally distributed enumeration variables were described with median and interquartile range, and Kruskal–Wallis rank-sum test was used for comparisons. Meanwhile, categorical variables are described by frequency and percentages, and chi-square test or Fisher exact probability method was used for comparison between groups. Spearman correlation, CHAID decision tree algorithm analysis, Multiple logistic regression, ordinal regression analysis, and receiver operating characteristic curve (ROC) was performed on SPSS (version 23.0, IBM, USA). Statistical tests were considered significant at *p* < 0.05.

## 3. Results

### 3.1. Incidence and mortality rates of Post-LT AKI in the cohort

Among the 272 patients with LT, the proportion of post-transplant AKI is 48.6% (131/272), of which the incidence of level 1 AKI is 42.0% (55/131), level 2 is 31.3% (41/131), and level 3 is 26.7% (35/131). In our cohort, the early post-operative mortality rate was10.3% (28/272), and accounting for 46.4% (13/28)of which were caused by AKI. The remaining reasons for post-operative death include post-operative infection, hemorrhage, and other individual patients with a poor prognosis due to pre-operative cardiovascular disease and endocrine system diseases. Moreover, among the patients with AKI, most patients who died in the early stages after LT belonged to grade 3 AKI (70%). Only a few patients died only with first or second-grade AKI, but their causes of death are often more complicated, and AKI was only a part of the factor.

### 3.2. Comparisons of clinical markers between AKI and non-AKI group

Table 1 shows the comparisons of the demographic data, pre-operative, intraoperative, and post-operative parameters between AKI and non-AKI groups in the model group. In pre-operative markers, decompensated liver cirrhosis, pretransplant AKI, baseline aspartate aminotransferase (AST), baseline total bilirubin (TB), Child-Pugh classification, and MELD scores were statistically significant between patients with AKI and non-AKI (all *p* < 0.05); Among intraoperative markers, the liver fatty of the donor, operation time, bleeding volume, blood transfusion volume, fluid balance, hypotension (SBP < 90 mmHg) occurrence and duration of hypotension, are statistically significant (All *p* < 0.05); For the post-operative parameters, the lactic acid immediately after the operation, ALT peak, AST peak, TBil peak, mechanical ventilation time, EAD, shock, infection, severe infection, ARDS, and re-ventilator number are statistically significant (All *p* < 0.05, Table 1). The parameters above are the risk factors of AKI after LT of the model group except for mechanical ventilation time and re-ventilator number, which may be the poor outcomes of post-LT AKI

**Table 1.** Comparison of basic information and clinical markers between AKI group and non-AKI group.

	AKI (*n* = 53)	non-AKI (*n* = 56)	*p*-Value
Genger (male)	44 (83.0%)	47 (83.9%)	0.551
Age (years)	54 ± 9	53 ± 10	0.350
BMI	24 ± 4	24 ± 3	0.946
Pretransplant parameters			
Primary liver disease			
Hepatoma	26 (49%)	7 (12.5%)	0.079
Acute/subacute liver failure	14 (26.4%)	7 (12.5%)	0.055
Decompensated liver cirrhosis	22 (41.5%)	11 (19.6%)	0.021*
Other primary diseases			
High blood pressure	10 (18.8%)	8 (14.2%)	0.463
Diabetes	10 (18.8%)	12 (21.4%)	0.190
Pretransplant infection	15 (28.3%)	12 (21.4%)	0.271
Pretransplant AKI	5 (9.4%)	0 (0%)	0.025*
Hepatorenal syndrome	2 (3.7%)	0 (0%)	0.234
Pre-operative serologic parameters			
Baseline creatinine (umol/L)	62 (52–73)	62 (53–67)	0.716
Baseline ALT (U/L)	38 (24–64)	34 (22–58)	0.436
Baseline AST (U/L)	58 (34–122)	41 (29–56)	0.009
Baseline TBil (μmol/L)	71 (27–238)	23.5 (17.8–61.8)	0.001*
Baseline serum sodium (mmol/L)	137 ± 5	137 ± 4	0.849
Child-Pugh classification^†^	–	–	0.002*
MELD sores	12 (7–22)	9 (6–13)	0.038*
Intraoperative parameters			
Fatty liver of donor	6 (11.3%)	1 (1.7%)	0.048*
Surgical pattern (classic orthotopic)	38 (71.6%)	42 (75.0%)	0.431
Cold ischemia time of donor liver (h)	5.0 (4.5–5.3)	5.0 (4.0–5.1)	0.112
Anhepatic phase time (min)	69 ± 14	65 ± 16	0.134
Operation time (h)	8.2 ± 1.2	7.3 ± 1.0	<0.001*
Intraoperative bleeding volume (ml)	1600 (1000–2500)	1000 (700–1600)	0.001*
Blood transfusion volume (ml)	1795 ± 1136	1319 ± 854	0.016*
Intraoperative fluid balance (ml)	4967 ± 2061	3882 ± 1449	0.002*
Intraoperative urine (ml)	1173 ± 490	1342 ± 462	0.069
Hypotension	34 (64.1%)	19 (33.9%)	0.001*
Hypotension time (min)	10 (0–20)	0 (0–5)	<0.001*
Post-transplant parameters			
The first post-operative lactic acid (mmol/L)	3.6 ± 2.1	2.7 ± 1.2	0.008*
ALT peak (U/L)	715 (421–1462)	471 (273–777)	<0.001*
AST peak (U/L)	1667 (943–3675)	913 (550–1556)	<0.001*
TBil peak (μmol/L)	130 (63–207)	58.0 (38.0–94.6)	<0.001*
EAD	25 (47.1%)	7 (12.5%)	<0.001*
ARDS	30 (56.6%)	7 (12.5%)	<0.001*
Mechanical ventilation time (h)	54 (24–92)	24 (12–36)	<0.001*
Re-ventilator	16 (30.1%)	3 (5.3%)	0.001*
Shock	18 (33.9%)	2 (3.5%)	<0.001*
Infection	41 (77.3%)	22 (39.2%)	<0.001*
Severe infections	21 (39.6%)	4 (7.1%)	<0.001*

AKI: acute kidney injury; BMI: Body Mass Index; ALT: alanine aminotransferase; AST: aspartate aminotransferase; TBIL: total bilirubin; MELD: model for end stage liver disease; EAD: early allograft dysfunction; ARDS: acute respiratory distress syndrome.

*p-value <0.05.

^†^Rank variable’s description part was omitted.

Enumeration variables were described as mean ± standard deviation (*SD*), and *t*-test was used for comparisons. Non-normally distributed enumeration variables were described with median (interquartile range), and Kruskal–Wallis rank sum test was used for comparisons.

### 3.3. Multivariate logistic regression analysis of AKI after LT

The above-mentioned risk factors with significant differences were included in the multivariate logistic regression. After excluding the confounding factor with a stepwise backward screening method, operation time (odds ratio [OR] = 2.125, *p* = 0.011), intraoperative hypotension time (OR = 1.090, *p* = 0.016) post-transplant AST Peak (OR = 1.001, *p* = 0.034), post-transplant infection (OR = 3.491, *p* = 0.031), post-transplant shock (OR = 7.159, *p* = 0.031) are independently associated with AKI after LT (Table 2). The logistic regression model established according to the results in Table 2, was then validated with the validation group. The ROC curve shows the good predictive ability of the model, with AUC = 0.755 (*p* < 0.001, 95% confidence interval [CI]: 0.701–0.809, Figure 3(M1)).

**Table 2.** Multivariate logistic regression analysis of post-transplant AKI.

	Log OR (β)	SE (β)	Wald value	*p*-Value	OR	95% CI (OR)
Operation time (h)	0.754	0.295	6.524	0.011	2.125	1.192–3.790
Intraoperative hypotension time (min)	0.086	0.036	5.837	0.016	1.090	1.016–1.169
The first post-operative lactic acid (mmol/L)	0.345	0.211	2.667	0.102	1.412	0.993–2.137
AST peak (U/L)	0.001	0.0003	4.513	0.034	1.001	1.000–1.001
Post-transplant infection (no = 0, yes = 1)	1.250	0.581	4.628	0.031	3.491	1.118–10.903
Post-transplant shock (no = 0, yes = 1)	1.968	0.913	4.649	0.031	7.159	1.196–42.846
Constant term	−9.506	2.570	13.683	0.000	0.002	–

Log OR (β): partial regression coefficient β; SE (β): standard error of β; OR: odds ratio; 95% CI (OR): 95% confidence interval of OR.

Additionally, to further facilitate clinical application, we established a risk score model based on logistics regression results. We stratified continuous variables and assigned scores for each stratification according to regression coefficients and reference value. The total score ranging from −2 to 34. Table 3 shows the prediction risk of AKI corresponding to each score. When the score ≥12, the probability of AKI is >99%, and when the score ≤0, the incidence of AKI is <50% (Table 3).

**Table 3.** Scoring model of post-transplant AKI risk prediction based on logistics regression.

Risk factor			Categories			|β|		Points
Operation time (h)						0.754		
			≤6.9					0
			7–7.9					3
			8–8.9					5
			9–9.9					7
			≥10					8
Intraoperative hypotension time (min)						0.086		
			0					−1
			1–9					0
			10–19					2
			20–29					5
			≥30					7
AST peak (U/L)						0.001		
			≤499					−1
			500–999					0
			1000–1499					1
			1500–1999					3
			2000–2999					5
			≥3000					11
Post-transplant infection (no = 0, yes = 1)						1.250		
			0					0
			1					3
Post-transplant shock (no = 0, yes = 1)						1.968		
			0					0
			1					5
Scoring system for AKI risk								
Point total	Estimate of risk	Point total		Estimate of risk	Point total		Estimate of risk	
−2	0.30	4		0.80	10		0.97	
−1	0.38	5		0.86	11		0.98	
0	0.47	6		0.90	12–34		0.99–1.00	
1	0.57	7		0.93				
2	0.66	8		0.95				
3	0.74	9		0.96				

### 3.4. Decision tree simplifies the prediction of post-transplant AKI predictors

**Figure 2.**
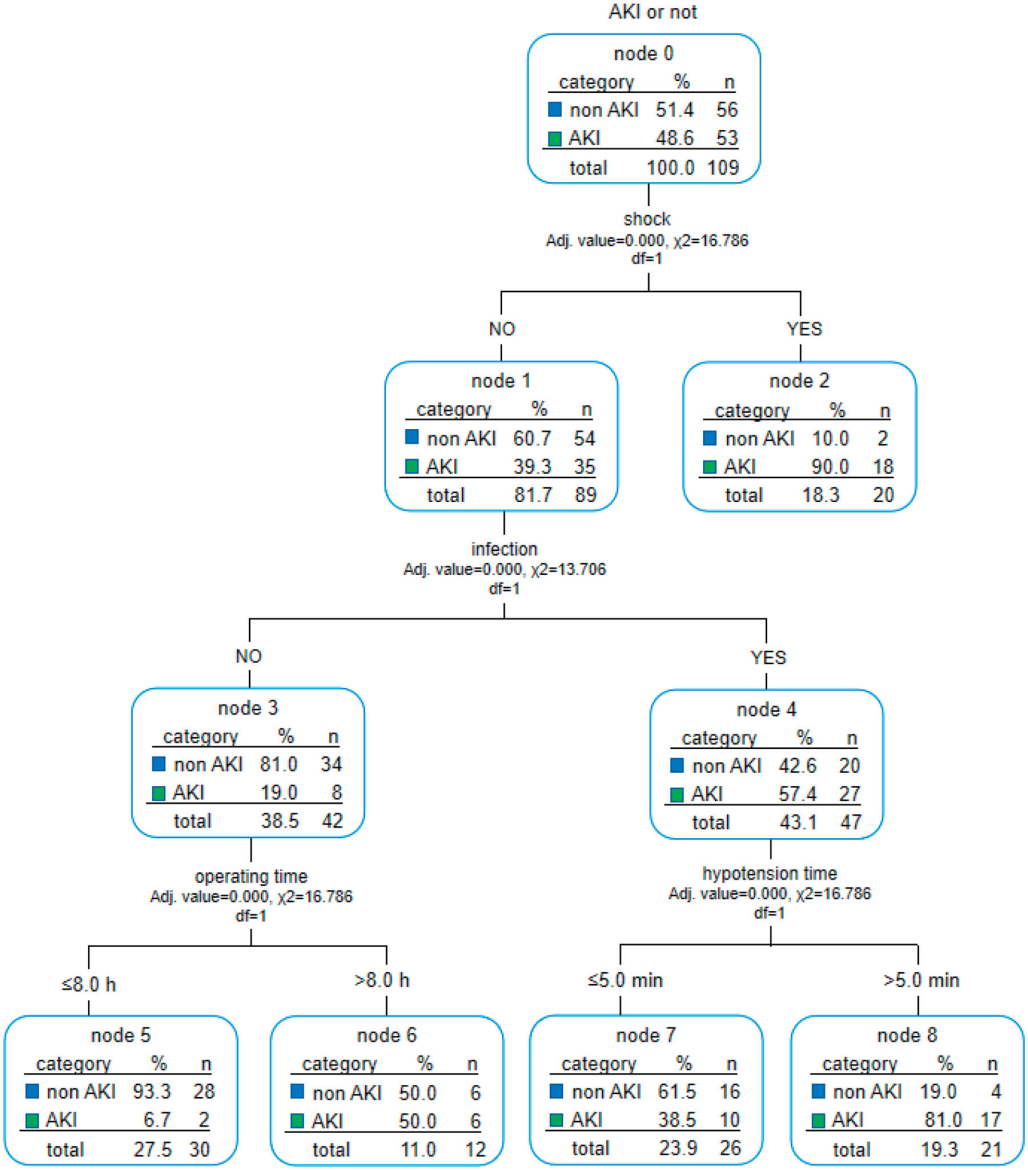
Decision tree for early detection of post-LT AKI. The two sets of numbers underneath each terminal node represent the proportions of AKI or non-AKI subjects. The subgroups were marked with green and blue according to prediction outcomes, e.g., subgroup 2 (node 2) represents the group with the predictive pattern for post-LT AKI (probability of AKI was 90%, green). The actual split values (thresholds) were indicated in the branches of the tree. LT: liver transplant; AKI: acute kidney injury; CART: classification and regression tree.

**Figure 3.**
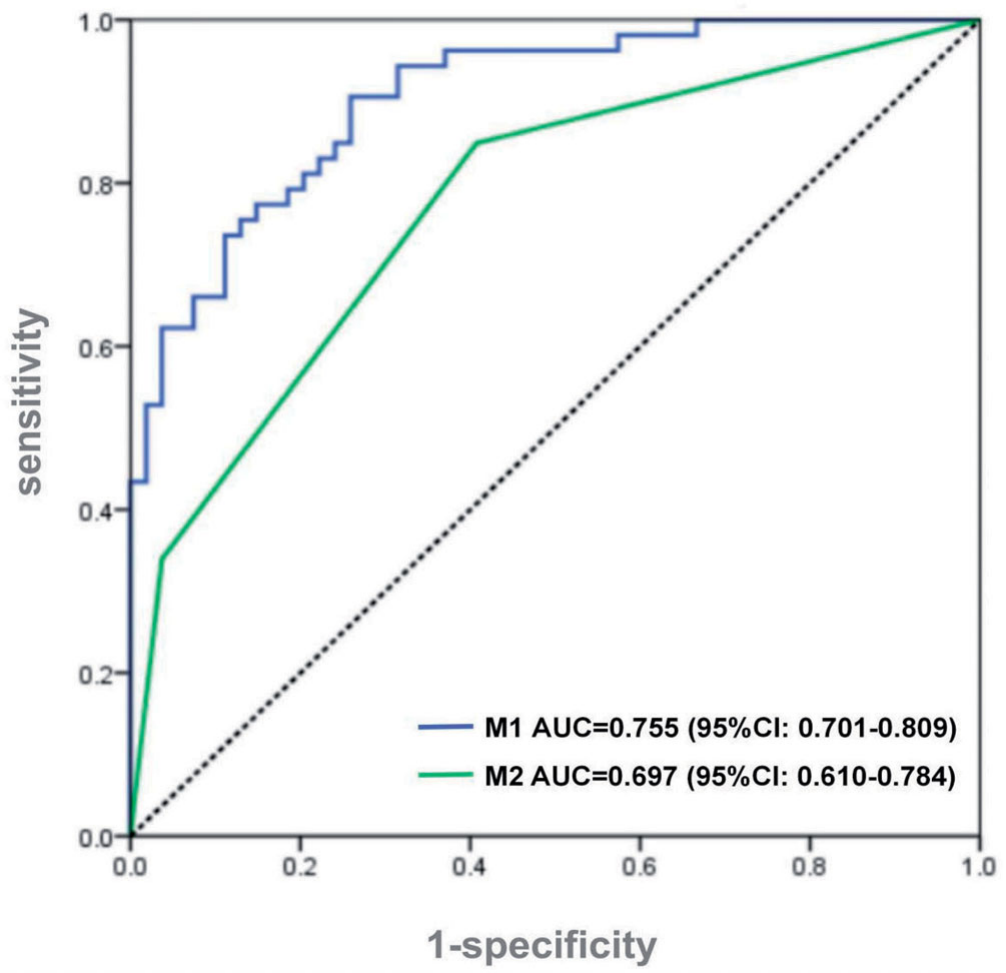
ROC curve of logistic regression scoring model and CART model. Both the scoring model (M1) and CART model (M2) worked well in detecting AKI patients after LT (AUC = 0.755 *vs.* 0.697). ROC: receiver operating characteristic curve; AUC: area under the curve; LT: liver transplant; AKI: acute kidney injury; CART: classification and regression tree.

To simplify the prediction method, we enrolled all the significant variables according to logistic regression analysis for CART analysis. The method of CHAID pre-pruning was used for pruning the tree, and the 10-fold cross-validation was used for internal validation of the tree (Figure 2). The tree shows that post-transplant shock is the root node of the tree, which indicates that shock is the most important discriminating factor with a proportion of 90% of patients with post-transplant shock diagnosed with AKI. Except for shock, post-transplant infection is the second biggest factor of AKI, and the incidence of AKI patients with infection is significantly higher than that without infection (57.4 vs. 19.0%). At the third level of the decision tree, hypotension time and operating time are the discriminating markers in patients with and without infection, respectively. And the tree also automatically provides the best cutoff values, 5 min and 8 h, respectively. Through cross-validation, the estimating risk is 0.275 of the model (*SD* = 0.043), and the prediction accuracy is 78%. In the validation cohort, the AUC of the ROC curve of the CART model was 0.697 (*p* < 0.001, 95% CI: 0.610–0.784, Figure 3(M2)).

## 4. Discussion

Early detection and treatment are crucial for the prognosis of patients with AKI after LT, especially early warning. The internationally recognized KDIGO criterion uses blood creatinine and urine volume for AKI diagnosis, but neither of them is the sensitive indicator that could reflect the decrease of glomerular filtration rate after LT [18,19], and resulting in a delay in the recognition of post-LT AKI and the inability in early treatment. It is necessary to establish an early detection model of post-LT AKI through some more sensitive clinical risk factors. In this study, we continuously enrolled 109 patients undergoing LT throughout 2019 (five patients with pre-transplant CKD were excluded) and divided them into AKI and non-AKI groups. By comparing the baseline information, pre-operative, intraoperative, and post-operative clinical markers, we identified the risk factors of AKI. In the following multiple logistic regression, operating time, hypotension time (>5 min), AST peak, post-transplant infection, and shock are independent risk factors for the post-transplant AKI, which are consistent with previous reports [5–7].

It was reported that hypotension during liver transplantation usually occurs in the reperfusion phase of liver transplantation, called post-reperfusion syndrome (PRS). Several studies have demonstrated that PRS was an independent risk factor for post-LT AKI [20,21], and was also demonstrated in our study that long hypotension during operation (>5 min) is an independent risk factor for post-LT AKI, which may be associated with PRS. PRS is defined as a decrease of >30% in mean arterial pressure (MAP) lasting at least 1 min within the first 5 min after reperfusion [22] and was also reported to be associated with post-LT AKI and post-LT AST peak level. Other studies showed that AST is also a sensitive indicator of ischemia-reperfusion injury, and the elevation of its peak is likely to cause by the warm ischemia, cold ischemia, and reperfusion of the donor's liver after transplantation [23]. All of the above illustrations indicate the important role of ischemia-reperfusion injury in the development of post-transplant AKI. That is to say, likely, any event that leads to inadequate renal perfusion during the perioperative period may promote the occurrence of AKI. On the other hand, a longer operation time could comprehensively reflect the prolongation of each surgical procedure, such as the cold ischemia phase and anhepatic phase. Similarly, infection is an important factor leading to post-operative hemodynamic instability and even septic shock and is also one of the most important risk factors for AKI. In addition, post-LT infection also leads to internal inflammatory reactions, which in turn causes further cell damage, especially kidney tubular damage.

We newly found that when we include all the indicators in the perioperative period (pre-operation, intraoperation, and post-operation) for logistic regression analysis, the pre-operative indicators had negligible risk compared with the intraoperative and post-operative indicators, and were automatically excluded in the statistical process. It indicated that compared with intraoperative and post-operative factors, pre-operative factors (such as MELD score, pre-operative liver, and kidney function, etc.) may not decisive in the development of post-LT AKI.

This study used Logistic regression to establish a more accurate multiple AKI risk scoring systems. We imitated Framingham's method of establishing integral models in the heart study. Through numerical calculations and model formulas, the statistically significant variables in the logistic analysis are assigned and their risks are calculated. The score ranges from −2 to 34, and the higher the score, the greater risk of AKI after LT. When the score ≥12, the risk of morbidity exceeds 99%. With a refined calculation, the integral model has strong operability but is not intuitive enough. On the other hand, the advantages of CART are manifested in two aspects [11]: ① It demonstrates the different degree of importance of each factor, such as post-transplant shock, infection, intraoperative hypotension time, and operation time on the development of AKI and the interaction between variables intuitively. For example, the root node of this decision tree model is post-transplant shock, which indicates that shock is the most important factor affecting the development of post-transplant AKI. It suggests that we should monitor the patient's blood pressure (BP) after surgery closely. If there is a drop in BP, it may be urgent for clinicians to find the cause and treat in time for avoiding the development of AKI, which may be more clinically practical than scoring system; ②The decision tree can automatically determine the optimal threshold for clinical application through strict binary calculation, and divide the candidate group into sub-groups with different degrees of risk set. For example, in this decision tree, individuals with surgery time ≥8 h and intraoperative hypotension time ≥5 min have a higher risk of AKI. So in general, CART is more convenient for clinicians to predict the development of AKI.

The limitations of our study include its retrospective in the validation group and some data were missing. For example, the usage and dosage of intraoperative vasoactive drugs were incomplete, although the main effect of vasoactive drugs on renal function is that they cause renal hypoperfusion, so there may be collinearity with intraoperative hypotension. In addition, it is necessary to conduct a multi-center and large-scale validation in the future to improve the models and further understand the occurrence and development of post-LT AKI and its mechanism.

In summary, by analyzing the factors before, during, and after liver transplantation, we identified 5 independent risk factors for the development of AKI following LT. They are operating time, intraoperative hypotension time, post-LT infection, the peak of post-transplant AST, and post-LT shock. These independent risk factors are all intraoperative and post-operative factors, rather than pre-operative factors, indicating that for the prevention of post-LT AKI, operation technique and post-operative management are more important, especially to ensure the stability of hemodynamics during intraoperative and post-operative and control the post-LT infection. Furthermore, the two prediction models, CHAID decision tree model, and the risk scoring system could complement each other, and describe the association between risk factors and AKI from different aspects, and provide intuitive and quantitative prediction tools for the prediction of post-LT AKI for clinical practice.

## Abbreviations

LT: liver transplant
AKI: acute kidney injury
RRT: renal replacement therapy
BMI: Body Mass Index
ALT: alanine aminotransferase
AST: aspartate aminotransferase
TBIL: total bilirubin
MELD: model for end stage liver disease
EAD: early allograft dysfunction
ARDS: acute respiratory distress syndrome
CKD: chronic kidney diseases
CART: classification and regression tree
CHAID: Chi-squared automatic interaction detector
ROC: receiver operating characteristic curve
AUC: area under the curve
